# Health-Related Quality of Life in Chinese Patients with Chronic Liver Disease

**DOI:** 10.1155/2012/516140

**Published:** 2012-06-03

**Authors:** Ru Gao, Feng Gao, Guang Li, Jian Yu Hao

**Affiliations:** Digestive Department, Beijing Chao-Yang Hospital, Capital Medical University, Chao Yang District, Beijing 100020, China

## Abstract

*Aim*. To investigate the factors contributing to health-related quality of life (HRQOL) in Chinese patients with chronic liver disease (CLD). *Methods*. HRQOL was measured with SF-36v2 Chinese version. Demographic and clinical data were collected, and patients with liver cirrhosis were divided into Child's Class A, B, and C according to Child-Turcotte-Pugh scoring system. *Results*. A total of 392 Chinese patients with CLD and 91 healthy controls were enrolled. HRQOL in patients with CLD was lower than that in healthy controls. Score of PCS in healthy controls was 54.6 ± 5.5 and in CLD was 47.8 ± 8.8 (*P* = 0.000). Score of MCS in healthy controls was 56.4 ± 8.1 and in CLD was 51.7 ± 7.4 (*P* = 0.000). Increasing severity of CLD from no cirrhosis to advanced cirrhosis was associated with a decrease on all domains of the SF-36 (*P* < 0.05). Stepwise linear regression analysis showed that severity of disease, age, present ascites, present varices, and prothrombin time had significant effect on physical health area. Severity of disease, female, present varices, total bilirubin, prothrombin time, and hemoglobin had significant effect on mental health area. *Conclusions*. Patients with CLD had impaired HRQOL. Increasing severity of CLD was associated with a decrease on HRQOL. Old age, female gender, advanced stage of CLD, present ascites, hyperbilirubinemia, and prolonging prothrombin time were important factors reducing HRQOL.

## 1. Introduction

Chronic liver disease (CLD) is increasing being recognized as an important cause of chronic disease worldwide because of its epidemiological burden, its potential impact on the patient's health, and their health-related quality of life (HRQOL) [1–4]. Chronic liver disease is also one of the most common chronic diseases in China, which is responsible for thousands of dollars per patient per year in direct and indirect medical costs [5, 6]. Patients with chronic liver disease suffer from fatigue, loss of self-esteem, inability to function at work, anxiety, depression, and other emotional problems that profoundly decrease their quality of life and well-being [7–13]. Many studies on HRQOL in Chinese patients with CLD are focused on chronic virus hepatitis B [12–14] and few studies on factors contributing to HRQOL in Chinese patients with CLD. Therefore, we aimed to investigate factors contributing to HRQOL in Chinese patients with CLD.

## 2. Methods

### 2.1. Ethics

The study received ethics approval from the Ethics Board of Beijing Chao-Yang Hospital, Capital Medical University, and all participants gave written informed consent.

### 2.2. Patient Selection

Between November 2009 and November 2010, all chronic liver disease eligible patients, age 18 to 80 years, both male and female, from the Liver Research Center of Beijing Friendship Hospital and the Digestive Department of Beijing Chao-Yang Hospital, Capital Medical University, were approached for participation in the study. Patients with other chronic active medical (such as congestive heart failure and chronic obstructive pulmonary disease) or psychiatric conditions, malignancy, a transplanted liver, and those unable to communicate or who declined to participate were excluded. The healthy controls were people without any chronic diseases, age 18 to 80 years, both male and female, who regularly took health checkup in our hospital's health examination centre.

Each patient had an established diagnosis by a hepatologist. And the diagnosis of chronic viral hepatitis B was based on the presence of hepatitis B surface antigen for more than six months, elevated serum alanine aminotransferase levels, with or without HBV DNA as detected by the hybridization method [15]. The diagnosis of chronic viral hepatitis C was based on a positive hepatitis C antibody (ELISA II analysis), elevated serum alanine aminotransferase levels, with or without HCV RNA as detected by polymerase chain reaction [16]. For both HBV and HCV, patients receiving interferon therapy in the previous 3 months were also excluded. The diagnosis of primary biliary cirrhosis was based on positive antimitochondrial antibody test results and elevated liver enzymes, with or without liver biopsy [17]. Alcohol was the etiology of chronic liver disease if daily alcohol drinking was greater than 40 g for at least 10 years, with elevated *γ*-glutamyl transferase, and ruled out the possibility of other liver diseases [18]. The diagnosis of autoimmune hepatitis was based on a simplified diagnostic criterion [19]. The diagnosis of liver cirrhosis was based on clinical, biochemical, serologic, ultrasonographic, and radiographic parameters. Clinical and laboratory assessment was conducted to measure the severity of liver cirrhosis using the Child-Turcotte-Pugh scoring system [20, 21]. The Child-Turcotte-Pugh scoring system included serum concentrations of total bilirubin and albumin, prothrombin time, the presence/absence of ascites and encephalopathy.

### 2.3. Data Collection

At admission, each patient gave his or her informed consent and then completed the self-administered HRQOL questionnaire: the Medical Outcomes Study of Short Form36 (SF-36 v2 Chinese version), a widely used and validated generic HRQOL questionnaire. Extensive demographic and clinical data were also collected at this time. Marital status was dichotomized into single and paired. Single was extended to include unmarried person, divorced, or deceased couple.

Laboratory data included alanine and aspartate aminotransferases, alkaline phosphatase, *γ*-glutamyl transferase, total bilirubin, serum albumin, serum creatine, prothrombin time, serum potassium and sodium, hemoglobin, white blood cell, and platelet counts (DADE Dimension Rx1 full-automatic biochemical analyzer).

### 2.4. HRQOL Survey

The SF-36 v2 Chinese Version (from Quality Metric Incorporated) consists of 36 items divided into eight domains that can be aggregated into two summary scores, a mental component summary (MCS) and a physical component summary (PCS). These domains range from those reflecting predominantly physical wellness, including physical function (PF), the ability to perform expected physical roles (RP), the degree of bodily pain (BP), and the overall sense of general health (GH), to those reflecting predominantly social and emotional well-being including overall sense of vitality (VT), ability to function in social roles (SF), ability to perform expected emotional and social roles (RE), and overall sense of mental health (MH). Scores for these eight SF-36 domains range between 0 and 100, whereas the summary scores for the physical component range between 8 and 73 and those for the mental component range between 10 and 74 [22–24].

### 2.5. Comparison Groups

There were two groups in our study: CH (healthy control) and CLD (chronic liver disease). As far as etiologies of CLD were concerned, there were six groups: AIH (autoimmune hepatitis), ALD (alcoholic liver disease), CHB (chronic viral hepatitis B), CHC (chronic viral hepatitis C), PBC (primary biliary cirrhosis), and unknown group. And patients with CLD were divided into no cirrhosis group, CA (Child's Class A), CB (Child's Class B), and CC (Child's Class C).

### 2.6. Statistical Methods

All data were analyzed with SPSS for Windows version 11.0. Categorical data were described as number and continuous data as mean ±SD, which were analyzed using independent sample *t* test or Chi-square test if suitable. A univariate analysis of general linear model was used to compare the severity of chronic liver disease on the domains of SF-36, in which the dependent variables were SF-36 domains, fixed factor was the group, and covariates were age and educational level. Stepwise lineal regression analysis was performed to study the influence of independent variables on domains of SF-36 while controlling the effect of other variables, in which the dependent variables were domains of SF-36; independents were age, gender, educational level, marital status, ascites, digestive bleeding, varices, and other clinical factors. A *P* value < 0.05 was considered as statistically significant.

## 3. Results

### 3.1. Demographic and Clinical Data of Respondents

A total of 392 Chinese patients with CLD and 91 healthy controls were enrolled in the study and completed the SF-36 v2. The demographic and clinical data were shown in Table 1. There were no statistically significant differences between the group of healthy control and CLD on age, gender, marital status, and educational level (*P* > 0.05).

Different etiologies of chronic liver disease were AIH (34 cases), ALD (112 cases), CHB (105 cases), CHC (63 cases), PBC (64 cases), and unknown (14 cases). Different severities of chronic liver disease were no cirrhosis (186 cases), CA (49 cases), CB (97 cases), and CC (60 cases).

### 3.2. Quality of Life in Patients with Chronic Liver Disease

Table 2 showed scores of SF-36 and clinical data between healthy control and chronic liver diseases groups. Except BP, other scores of SF-36 were statistically significantly reduced in chronic liver diseases group. Patients with chronic liver diseases showed statistically significantly decreasing level of albumin, white blood cell, hemoglobin, and platelet. And patients with chronic liver diseases also showed statistically significantly increasing level of alanine aminotransferase, aspartate aminotransferase, alkaline phosphatase, *γ*-glutamyl transferase, total bilirubin, blood urea nitrogen, and prothrombin time.

Table 3 showed increasing severity of CLD from no cirrhosis to advanced cirrhosis (Child's Class A, Child's Class B, to Child's Class C) was associated with statistically significant decreasing on all scores of SF-36. Pairwise comparisons between groups showed scores of Child's Class C group were statistically significantly reduced.

### 3.3. The Effect of Demographic and Clinical Characteristics on Quality of Life

Table 4 showed the results of stepwise linear regression analysis of different demographic and clinical data on scores of SF-36. The demographic and clinical data in the stepwise linear regression analysis explained 0.518 and 0.152 on PCS and MCS scores of SF-36 as indicated by the *R* square. Take PCS score; for example, age, disease severity, present ascites, and prothrombin time had negative effect on PCS. Our model predicted decreasing score of PCS by 0.129 each increasing year of life, decreasing score of PCS by 0.385 each prolonging 1 second of prothrombin time, decreasing score of PCS by 0.385 in patients with present ascites, decreasing score of PCS by 2.333 each progressing degree of disease (from no cirrhosis, Child's Class A, Child's Class B to Child's Class C).

## 4. Discussion

We found that patients with chronic liver disease had impaired HRQOL compared with healthy people, which agreed with many studies [2–4, 7, 12–15]. Similar scores on BP resulted from similar scores among healthy control, no cirrhosis, Child's A and Child's B groups only Child's C group's scores of BP were significantly reduced. Our data also found that increasing severity of chronic liver disease from no cirrhosis to advanced cirrhosis (Child's Class C) was associated with a decrease on HRQOL. Other recent publications also showed that chronic liver diseases substantially reduced HRQOL; this impact did not differ markedly by type of disease, and increasing disease severity was associated with poorer HRQOL, especially relating to a physical component [2–4, 10–13, 25–31]. With the progressing of liver dysfunction, patients with CLD suffer from fatigue, loss of self-esteem, inability to function at work, anxiety, depression, and other emotional problems that profoundly decrease their quality of life and well-being [22, 32]. Patients with liver cirrhosis also suffer from complications, which will reduce their HRQOL, especially on physical domain area for their difficulty to maintain daily work and life [22–24, 32–36].

Our study evaluated the factors contributing to HRQOL in Chinese patients with CLD, such as age, gender, marital status, educational level, and laboratory data. Stepwise linear regression analysis showed that increasing disease severity reduced patients' PF, RP, GH, VT, SF, and PCS. Aging had negative effect on multiple domains, especially on physical domains. And female gender had negative effect on multiple domains, especially on mental domains; possible cause was that female patients paid more attention on their health and spent more time on consulting treatment. Marital status had positive effect on GH, because married patients with CLD could get psychological support from their partner. Present ascites had negative effect on patients' PF and PCS, for patients with ascites finding it difficult to maintain daily work and life. And patients with varices lose function to do daily work and had impaired RP and RE. Level of hemoglobin had positive effect on SF and MH because patients with normal hemoglobin could take part in social activities (visiting relatives and friends). BUN also had positive effect on MCS. Liver failure can cause malabsorption of nutrition, insufficient intake, and massive consumption of protein, which cause reduced BUN and negative nitrogen balance, while high value of BUN within normal range suggested positive nitrogen balance and relatively better liver function. Prolonged prothrombin time had negative effect on multiple domains of HRQOL because patients with poor prothrombin function reduced their ability on physical, social, and mental domains. Recent studies [22, 25, 36–41] supported our findings that aging, female gender, present ascites, and prolonging prothrombin time had negative effect on HRQOL. Potential treatable factors [42–44], correction of ascites, hypoalbuminemia, minimal hepatic encephalopathy, and anemia, eating BCAA-enriched snack and long-term late-evening snack may cause a positive impact on HRQOL in patients with CLD.

We also found etiology had no effect on HRQOL in patients with CLD. But some researches [11, 12, 37, 45] suggested that patients with primary biliary cirrhosis had impaired HRQOL than other liver diseases. However, we got support from researches [25–29] that different etiologies of liver disease had similar HRQOL.

There are two basic types of HRQOL questionnaires, which measure HRQOL from the patient perspective: generic questionnaires and disease-specific questionnaires. A third type of HRQOL questionnaire exists from a cost-effectiveness perspective, called utility measures. In this study, we used SF-36v2, a widely used generic questionnaire, to measure the HRQOL in Chinese patients with chronic liver disease and to compare it with Chinese healthy controls. SF-36 is a short and easy questionnaire; it only takes patients several minutes to complete and can be used in developing countries and provide information that is complementary to the clinical data. HRQOL measures, such as SF-36, can help to integrate the biomedical and the psychosocial models of health. This integrated approach to the study of chronic liver disease will capture the impact of these diseases on patients' health and well-being [22–24, 32–36].

Our study has some limitations. All subjects were recruited from two hospitals in one city, which might have potential selection bias. Our study included patients with a wide spectrum of disease severity (from no cirrhosis to Child's Class C) and different etiologies (AIH, ALD, CHB, CHC, and PBC), and the number of patients is relatively less. We are addressing these issues in ongoing studies and will assess more patients in more centers, explore SF-36 in more patients with CLD, and take into account the economic income and burden of the patients. And we also want to reevaluate the HRQOL after medical treatment.

In summary, patients with CLD had impaired HRQOL, and increasing severity of CLD was associated with a decrease on HRQOL. Old age, female gender, advanced stage of CLD, present ascites, hyperbilirubinemia, and prolonging prothrombin time were important factors reducing HRQOL.

## Figures and Tables

**Table 1:** Demographic data of different groups.

Items	Healthy control	Chronic liver disease				Independent sample *t* test or
	*n* = 91	*n* = 392				Chi-square
Age (mean ± SD, yr)	53.4 ± 12.7	53.8 ± 13.4				*P* = 0.806
Male/female (*n*)	57/34	229/163				*P* = 0.461
Paired/single (*n*)	88/3	374/18				*P* = 0.585
Educational level (mean ± SD, yr)	11.5 ± 3.4	11.2 ± 3.2				*P* = 0.408
Etiologies of chronic liver disease	—	No cirrhosis	Cirrhosis			—
			CA	CB	CC	
AIH	—	17	2	8	7	—
ALD	—	61	5	25	21	—
CHB	—	49	17	20	19	—
CHC	—	28	11	19	5	—
PBC	—	31	11	19	3	—
Unknown	—	—	3	6	5	—
Present ascites	—	143				—
Present varices	—	181				—

AIH: autoimmune hepatitis, ALD: alcoholic lLiver disease, CHB: chronic hepatitis B, CHC: chronic hepatitis C, PBC: primary biliary cirrhosis. CA: cirrhosis Class A, CB: cirrhosis Class B, CC: cirrhosis Class C.

**Table 2:** HRQOL and clinical data of different groups.

Item	Healthy control	Chronic liver disease	Independent sample *t* test
	*n* = 91	*n* = 392	
Physical function	92.5 ± 6.6	80.6 ± 21.6	*P* < 0.001
Physical roles	93.0 ± 11.8	73.7 ± 24.2	*P* < 0.001
Bodily pain	86.9 ± 15.5	83.8 ± 19.0	*P* = 0.108
General health	76.2 ± 18.0	49.9 ± 23.2	*P* < 0.001
Vitality	83.4 ± 11.8	73.4 ± 14.6	*P* < 0.001
Social roles	94.2 ± 10.4	77.6 ± 22.9	*P* < 0.001
Emotional roles	92.7 ± 10.8	86.9 ± 17.4	*P* < 0.001
Mental health	87.5 ± 24.5	77.2 ± 14.1	*P* < 0.001
PCS	54.6 ± 5.5	47.8 ± 8.8	*P* < 0.001
MCS	56.4 ± 8.1	51.7 ± 7.4	*P* < 0.001
Laboratory data			
Albumin (g/L)	36.3 ± 2.8	33.3 ± 7.8 (*n* = 330)	*P* < 0.001
ALT (U/L)	22.5 ± 9.1	53.2 ± 60.8 (*n* = 328)	*P* = 0.006
AST (U/L)	22.5 ± 10.7	66.7 ± 71.9 (*n* = 328)	*P* < 0.001
GGT (U/L)	49.5 ± 51.6	101.6 ± 163.7 (*n* = 323)	*P* < 0.001
ALP (U/L)	99.1 ± 8.1	127.3 ± 88.9 (*n* = 323)	*P* = 0.001
TBIL (mg/dL)	0.57 ± 0.27	1.96 ± 2.58 (*n* = 323)	*P* < 0.001
BUN (umol/L)	5.1 ± 0.9	6.0 ± 3.9 (*n* = 329)	*P* < 0.001
K (umol/L)	4.00 ± 0.28	4.03 ± 0.48 (*n* = 329)	*P* = 0.531
Na (umol/L)	140.2 ± 3.41	146.1 ± 8.63 (*n* = 329)	*P* = 0.092
Prothrombin Time (s)	10.4 ± 0.4	14.2 ± 3.8 (*n* = 329)	*P* < 0.001
WBC (10^9^/L)	5.93 ± 1.56	4.72 ± 2.69 (*n* = 330)	*P* < 0.001
Hemoglobin (g/L)	138.1 ± 22.2	113.6 ± 27.4 (*n* = 330)	*P* < 0.001
Platelet (10^9^/L)	206.0 ± 44.3	115.6 ± 69.2 (*n* = 330)	*P* < 0.001

PCS: physical component summary, MCS: mental component summary ALT: alanine aminotransferase, AST: aspartate aminotransferase, ALP: alkaline phosphatase, GGT: *γ*-glutamyl transferase, TBIL: total bilirubin, BUN: blood urea nitrogen, K: serum potassium, Na: serum sodium, WBC: white blood cell.

**Table 3:** HRQOL scores of patients with different severities of CLD (mean ± SD).

SF-36 Dimension	No cirrhosis	CA	CB	CC	Univariate aAnalysis of variance
	*n* = 186	*n* = 49	*n* = 97	*n* = 60	
PF	91.7 ± 9.3^c,d^	85.2 ± 17.8^c,d^	72.4 ± 21.5^a,b,d^	55.7 ± 25.9^a,b,c^	*P* < 0.001
RP	88.2 ± 13.2^b,c,d^	73.3 ± 22.2^a,c,d^	61.6 ± 21.5^a,b,d^	48.7 ± 25.8^a,b,c^	*P* < 0.001
BP	88.5 ± 13.6^d^	85.8 ± 17.4^d^	83.5 ± 19.4^d^	68.3 ± 25.2^a,b,c^	*P* < 0.001
GH	63.5 ± 18.5^b,c,d^	55.1 ± 20.1^a,c,d^	35.1 ± 16.7^a,b^	29.0 ± 18.3^a,b^	*P* < 0.001
VT	79.0 ± 9.5^b,c,d^	74.3 ± 13.1^a,d^	70.8 ± 13.1^a,d^	59.3 ± 20.1^a,b,c^	*P* < 0.001
SF	89.1 ± 14.1^c,d^	83.1 ± 18.4^c,d^	66.2 ± 15.8^a,b,d^	55.2 ± 25.1^a,b,c^	*P* < 0.001
RE	92.4 ± 9.8^c,d^	86.2 ± 15.8^d^	84.6 ± 17.9^a,d^	74.2 ± 26.8^a,b,c^	*P* < 0.001
MH	78.4 ± 10.6^d^	80.1 ± 11.3^d^	79.2 ± 12.8^d^	67.8 ± 21.8^a,b,c^	*P* < 0.001
PCS	53.1 ± 5.9^b,c,d^	48.7 ± 6.6^a,c,d^	42.4 ± 7.1^a,b,d^	37.2 ± 8.9^a,b,c^	*P* < 0.001
MCS	53.2 ± 4.8^d^	52.7 ± 6.5^d^	51.8 ± 7.5^d^	46.8 ± 7.5^a,b,c^	*P* < 0.001

Mean age = 53.83, mean educational level = 11.19. CLD: chronic liver disease, CA: cirrhosis Class A, CB: cirrhosis Class B, CC: cirrhosis Class C, PF: physical function, RP: physical roles, BP: bodily pain, GH: general health, VT: vitality, SF: social roles, RE: emotional roles, MH: mental health, PCS: physical component summary, MCS: mental component summary, SF-36: 36-item short form. Pairwise comparisons: ^a^ *P* < 0.05 versus no cirrhosis, ^b^ *P* < 0.05 versus CA, ^c^ *P* < 0.05 versus CB, ^d^ *P* < 0.05 versus CC.

**Table 4:** Results of stepwise linear regression analysis.

Item	SF-36 Dimension and unstandardized coefficients (*n* = 411)									
	PF	RP	BP	GH	VT	SF	RE	MH	PCS	MCS
Constant	137.773	124.686	113.618	70.895	92.104	92.052	99.491	103.914	67.407	59.778
Age	−0.398	−0.247	−0.140	—	—	−0.161	−0.181	—	−0.129	—
Gender	−3.216	—	—	—	−3.023	—	—	−4.295	—	−1.673
Married	—	—	—	13.628	—	—	—	—	—	—
Disease Severity	−2.912	−6.016		−11.670	−4.111	−5.881	—	—	−2.333	—
Ascites	−7.616	—	—	—	—	—	—	—	−3.001	—
Varices	—	−6.389	—	—	—	—	−4.370	—	—	—
BUN	—	—	—	—	—	—	—	—	—	0.330
Hemoglobin	—	—	—	—	—	0.136	—	—	—	0.312
Etiology	—	—	—	—	—	—	—	—	—	—
PT	−1.488	−1.253	−1.681	—	—	−0.999	−1.209	−1.238	−0.385	−0.767
K	—	—	—	—	—	—	3.984	—	—	—
Na	—	—	—	—	—	—	—	—	—	—
TBIL	—	—	—	—	−1.147	—	—	−0.894		—
Albumin	—	—	—	—	—	—	—	—	—	—
ALT	—	—	—	—	—	—	—	—	—	—
AST	—	—	—	—	—	—	—	—	—	—
GGT	—	—	—	—	—	—	—	—	—	—
ALP	—	—	—	—	—	—	—	—	—	—
WBC	—	—	—	—	—	—	—	—	—	—
Platelet	—	—	—	—	—	—	—	—	—	—
R square	0.458	0.438	0.121	0.460	0.256	0.375	0.181	0.111	0.518	0.152

BUN: blood urea nitrogen, PT: prothrombin time, K: serum potassium, Na: serum sodium, TBIL: total bilirubin, ALT: alanine aminotransferase, AST: aspartate aminotransferase, ALP: alkaline phosphatase, GGT: *γ*-glutamyl transferase, WBC: white blood cell. PF: physical function, RP: physical roles, BP: bodily pain, GH: general health, VT: vitality, SF: social roles, RE: emotional roles, MH: mental health, PCS: physical component summary, MCS: mental component summary, SF-36: 36-item short form. Only data with *P* < 0.05 were expressed as values of beta-coefficients. “—”: *P* > 0.05.
